# Clinical efficacy of electroacupuncture for urinary incontinence following spinal cord injury: a meta-analysis and trial sequential analysis

**DOI:** 10.3389/fneur.2025.1573090

**Published:** 2025-09-26

**Authors:** Ping-Yan Zhang, Chuang-Long Xu, Ga-Xi Ye, Lu Meng, Feng Wang

**Affiliations:** ^1^Ningxia Hui Autonomous Region Hospital of Traditional Chinese Medicine, Ningxia Hui Autonomous Region Academy of Traditional Chinese Medicine, Yinchuan, Ningxia, China; ^2^General Hospital of Ningxia Medical University, Yinchuan, Ningxia, China

**Keywords:** electroacupuncture, spinal cord injury, urinary incontinence, meta-analysis, trial sequential analysis

## Abstract

**Purpose:**

Urinary incontinence (UI) is a prevalent clinical manifestation in spinal cord injury (SCI) patients, occurring in approximately 70% of these individuals. This systematic review aims to comprehensively evaluate the research evidence on electroacupuncture (EA) for UI after SCI, assess its clinical efficacy and safety, and provide a reference for clinical practice.

**Method:**

Eight databases were searched for randomized controlled trials (RCTs) published from inception to May 20th, 2025. RCTs comparing EA (with or without conventional rehabilitation, CR) to CR alone for managing UI after SCI were included. Data were analyzed using R version 3.6.3. In accordance with PRISMA-2020 guidelines, two reviewers independently extracted data and assessed the risk of bias using the Cochrane risk of bias tool (ROB 2.0). The certainty of the evidence was graded using the GRADE (Grading of Recommendations Assessment, Development, and Evaluation) according to GRADE handbook.

**Results:**

A total of 15 studies were included, comprising 1,394 patients with UI after SCI. The meta-analysis indicated that, compared to the CR group, the EA group showed a significant improvement in 24 h incontinence frequency (MD = −1.42, 95% CI [−1.88, −0.96], *p* < 0.01), maximum urine output in 24 h (MD = 18.98, 95% CI [9.27, 28.69], *p* < 0.01), and single urination volume in 24 h (MD = 30.76, 95% CI [21.45, 40.08], *p* < 0.01). Regarding the Urodynamic outcome indices, the EA group displayed significant improvement in residual urine volume (MD = −20.06, 95% CI [−28.73, −11.38], *p* < 0.01), bladder volume (MD = 38.86, 95% CI [19.98, 57.75], *p* < 0.01), maximum urine flow rate (Qmax) (MD = 2.68, 95% CI [1.66, 3.70], *p* < 0.01), detrusor pressure (PdetQmax) (MD = −6.77, 95% CI [−9.54, −4.00], *p* < 0.01), and bladder compliance (BC) (MD = 1.41, 95% CI [0.88, 1.93], *p* < 0.01). Trial Sequential Analysis (TSA) confirmed the superior treatment outcomes of EA compared to CR. The reported adverse events related to acupuncture were minimal and less severe.

**Conclusion:**

EA exhibits considerable potential to enhance self-control of bladder function in patients with UI following SCI. However, this study has certain limitations, and higher quality randomized controlled trials are necessary to confirm these findings.

**Systematic review registration:**

https://www.crd.york.ac.uk/prospero/, identifier CRD42024594516.

## 1 Introduction

The spinal cord functions as both the neural pathway and the central hub for urinary reflexes. After spinal cord injury (SCI), a significant majority of patients experience urinary dysfunction, characterized by reduced self-control over urination and impaired regulation of urinary movements. Urinary incontinence (UI) is a prevalent clinical manifestation in SCI patients, affecting approximately 70% individuals (1). Bladder detrusor hyperactivity and abnormal urethral sphincter contractions following SCI are major contributing factors to UI (2).

UI not only imposes substantial inconvenience on patients’ lives but also poses a risk for various complications (3, 4), including physical, social, and emotional impairments, and an increased risk of febrile urinary tract infections. Effective bladder management is crucial to prevent these infections (5, 6). Traditional approaches for the treatment of urinary incontinence caused by neurological disorders include pharmacological therapies, surgical interventions, and behavioral training (7). Alternative therapies encompass physical neuromodulation techniques and integrative approaches combining traditional Chinese and Western medicine (such as electroacupuncture of the pudendal nerve). The latter has been shown to significantly improve bladder dysfunction associated with conditions like multiple sclerosis by enhancing pelvic floor muscle contraction (8).

Acupuncture, as a complementary alternative therapy, offers certain advantages in alleviating incontinence symptoms (9, 10). In this context, Electroacupuncture (EA) refers to the technique in which pulse current is applied after elicitation of “Deqi” via filiform needle insertion, modality that integrates traditional acupuncture with modern electrical stimulation techniques, holds promise for the treatment of UI following SCI patients (11). EA stimulation may induce electrophysiologic changes and modulate neurotransmitter activity in the bladder, exhibiting both excitatory and inhibitory effects (12, 13). This dual modulation can enhance voiding function by improving the contraction of a weakened detrusor muscle and inhibiting hyperreflexia, thus enhancing the bladder’s storage function (14). Additionally, EA can regulate the coordination between the bladder detrusor and urethral sphincter.

Recent research has increasingly focused on the effects of EA on UI following SCI patients (15–17). And this systematic review aims to comprehensively evaluate on the efficacy and safety of EA for UI following SCI and provide guidelines for clinical practice. We will employ trial sequential analysis (TSA) to assess whether the included trials have reached the optimal information size and if the cumulative data are adequately powered to evaluate outcomes.

## 2 Materials and methods

### 2.1 Literature search strategy

We registered the protocol on the PROSPERO (ID: CRD42024594516) and conducted our study in accordance with the Preferred Reporting Items for Systematic Reviews and Meta-analyses 2020 (PRISMA-2020) (18) guidelines in Supplementary material. We searched the following eight databases for Chinese and English articles from inception to May 20th, 2025: PubMed, Embase (Ovid), Web of Science, Cochrane Library, CBM, CNKI, Wanfang, and VIP. The search strategy details are provided in Supplementary Tabl S1.

### 2.2 Inclusion and exclusion criteria

All enrolled UI patients met the diagnostic criteria for SCI established by the American Spinal Injury Association, and had completed the spinal shock stage, neurogenic bladder was diagnosed as incontinence following spinal cord injury. The experimental group was treated with EA (involves piercing the skin and eliciting a deqi sensation), which can be supplemented with conventional rehabilitation (CR) measures. The CR including Western medicine, instrumental assistance, physical therapy. The control group was treated with CR measures or combined with sham acupuncture. The outcome measures included: urination diary indices (24 h incontinence frequency, 24 h maximum urine output, 24 h single urination volume) and urodynamic outcome indices [residual urine volume, bladder volume, maximum urine flow rate (Qmax), detrusor pressure (PdetQmax), bladder compliance (BC)].

Only RCTs that utilized EA for treating UI after SCI were included. We excluded articles that were not available in full text and other publication types such as letters, comments, and conference abstracts. Studies for which complete data could not be obtained or that used the same patient data as other included articles were also excluded. The eligible trials met the following PICOS (participants, interventions, comparisons, outcomes, and study design) criteria.

### 2.3 Data extraction

Two researchers independently selected the studies, collected the data, and imported the determined studies into EndNote 20. Any disagreements were resolved by a third researcher. Initially, articles with duplicate data were excluded. Subsequently, unrelated research was excluded based on the title and abstract. Then, the remaining studies were reviewed in detail to determine the final selection. Data for each included study were entered into Microsoft Excel (2016), including the study ID, age, sex, sample size, disease duration, intervention time, study design, acupuncture points and the outcomes.

### 2.4 Quality assessment

Two reviewers independently assessed the risk of bias for each included study using the Cochrane Risk of Bias (ROB) tool 2.0 (19). The certainty of the evidence was graded using the GRADE (Grading of Recommendations Assessment, Development, and Evaluation) according to (GRADE handbook). Disagreements between reviewers were resolved by a third researcher.

### 2.5 Strategy for data synthesis

Meta-analysis was conducted whenever outcomes were comparable across studies. Data analysis was performed using R 3.6.3. Continuous data were presented as mean difference (MDs) with 95% CI, and dichotomous data were presented as relative risk (RR) with 95% CI. Standardized mean differences (SMDs) with 95% CIs were calculated for studies using different outcome scales, and MDs with 95% CIs were calculated for studies using the same outcome scale (20). Heterogeneity was categorized as low (I^2^ < 50%), moderate (I^2^ = 50–74%), or high (I^2^ ≥ 75%) (21). Due to conceptual heterogeneity in acupuncture RCTs, a random effect model was used. Publication bias was assessed using Egger’s test when more than 10 studies were included in the analysis (22). If the heterogeneity was considerable, we would conduct subgroup analysis. The sensitivity analyses were conducted to assess robustness of the synthesized results. Trial sequential analysis (TSA) was conducted to determine if the optimal information size was reached and if the cumulative data were sufficiently powered to evaluate the outcomes. TSA software 0.9.5.10 beta (Copenhagen Trial Unit, Denmark) was used (23–25). An optimal information size was defined with a two -sided 5% risk of a type I error or a 20% risk of a type II error (80%power).

## 3 Results

### 3.1 Description of included trials

**Figure 1:**
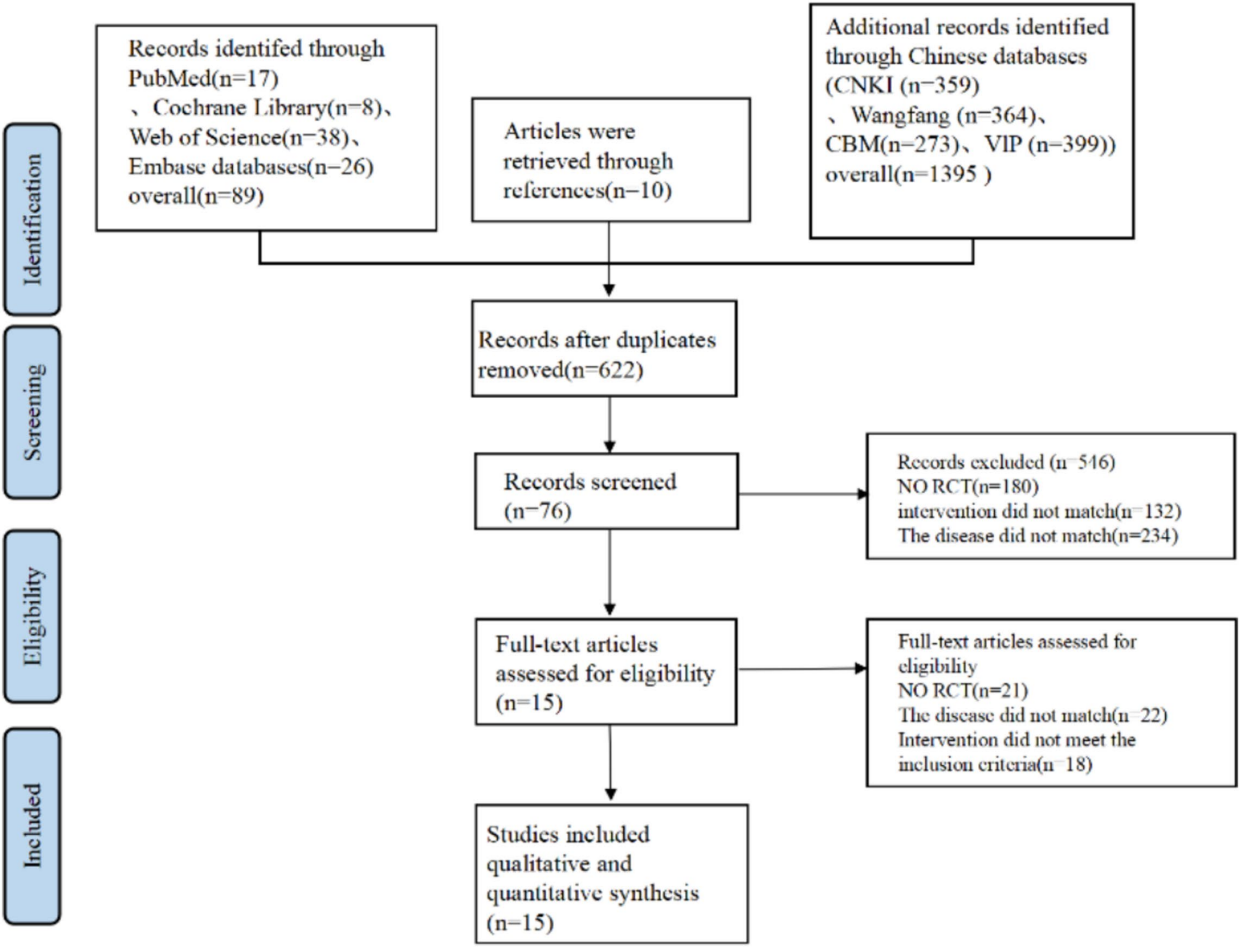
Process of identification and selection of relevant articles in this meta-analysis.

At first, a total of 1,484 articles were identified through database searches, with 89 from Pubmed, Cochrane, Embase, and Web of Science. 1,395 from CNKI, CBM, VIP, and Wan fang Data. Additional 10 records were identified through reference lists. After removing 862 duplicate articles, 622 articles underwent title and abstract screening. Of these, 546 articles were excluded due to lack of relevance, leaving 76 articles that met the inclusion criteria. Subsequently, 61 articles were excluded for the following reasons: not RCT (21 articles), disease mismatch (12 articles), patients mismatch (10 articles), and interventions not meeting inclusion criteria (18 articles). Ultimately, 15 RCTs (15–17, 26–37) were included (Figure 1).

All included studies were published and conducted in Chinese (Table 1). 14 studies employed a two-armed, one study employed a three-armed (35), parallel design, with sample sizes ranging from 35 to 164 participants. The included studies comprised 1,394 patients with a mean age ranging from 20 to 68 years. All diagnosed with UI following SCI. And all the studies included were uppermotor neuron injuries. The experimental interventions included EA, EA combined with conventional rehabilitation, while the control groups received conventional rehabilitation across all studies. Outcome measures included urination diary indices (24-h incontinence frequency, 24-h maximum urine output, 24-h single urination volume) and urodynamic indices [residual urine volume, bladder volume, maximum urine flow rate (Qmax), detrusor pressure (PdetQmax), bladder compliance (BC)].

**Table 1:** Information of the included studies.

First author (year)	ASIA	Disease course (month)		Age (years)		Sex (male/female)		Intervention		Acupuncture points	Course of treatment (d)	the timing of the first assessment	Outcomes
		Trial	Control	Trial	Control	Trial	Control	Trial	Control				
Feng 2014 (26)	B	4.62 ± 1.20	4.79 ± 1.62	29.8 ± 12.1	25.2 ± 10.8	16/7	14/6	EA + CR (*n* = 23)	CR (*n* = 20)	BL32, BL23, BL28	28	2011.01–2013.06	c,d,f
Meng 2015 (34)	NR	12.9 ± 3.8	13.5 ± 4.2	36 ± 8	36 ± 7	/	/	EA + CR (*n* = 20)	CR (*n* = 15)	CV3, CV4, BL23, BL35, BL31-34	28	2010.01–2013.12	a
Geng 2017 (27)	A58 B70	6.11 ± 2.37	6.56 ± 2.17	32.8 ± 8.55	34.2 ± 9.56	40/26	38/24	EA (*n* = 66)	CR (*n* = 62)	CV4	28	2015.02–07	e,f,g,h
Liu 2017 (28)	B	NR	NR	35.8 ± 10.2	33.6 ± 9.7	43/39	40/42	EA + CR (*n* = 82)	CR (*n* = 82)	CV3, CV4, CV6, ST25, EX-B2, BL31-34, BL35BL57, KI1	60	/	b,c,e,g,h
Jiang 2019 (30)	A29 B21	19.2 ± 4.7	19.8 ± 5.1	34.6 ± 6.5	35.1 ± 6.1	17/8	18/7	EA + CR (*n* = 25)	CR (*n* = 25)	CV3, CV4, SP6	28	2015.08–2017.08	d,e,f,g
Cheng 2019 (29)	NR	/	/	37 ± 11	33 ± 11	26/8	25/8	EA + CR (*n* = 34)	CR (*n* = 33)	BL31-34, BL35	30	2014.01–2016.10	d
Ke 2021 (31)	NR	1.50 ± 0.34	1.50 ± 0.34	36.31 ± 7.85	36.25 ± 7.78	28/14	27/15	EA + CR (*n* = 42)	CR (*n* = 42)	BL28, BL25, BL2, CV4, CV3, BL31-34, BL35	56	2017.10–2019.10	b,d,e
Wei 2021 (33)	B	2.12 ± 0.39	1.48 ± 0.36	43.48 ± 11.62	44.48 ± 10.79	15/6	16/5	EA + CR (*n* = 21)	CR (*n* = 21)	BL23, BL28, BL31-34, BL35	21	2017.01–2020.06	a,c,d,e,g
Li 2021 (32)	B	1.42 ± 0.67	1.58 ± 0.72	43.35 ± 5.72	42.15 ± 5.34	20/10	18/12	EA + CR (*n* = 30)	CR (*n* = 30)	CV4, CV3, SP6, KI6, CV2	42	2017.06–2020.06	a,c,d,e,g,h
Zhu 2022 (17)	B	2.09 ± 0.79	1.91 ± 0.61	44.41 ± 11.63	45.47 ± 10.34	21/11	22/10	EA + CR (*n* = 30)	CR (*n* = 30)	BL31-34	21	2019.01–2020.12	a,d,c,e,f
Zhang 2023 (16)	B	6.32 ± 1.96	5.75 ± 1.87	47.45 ± 6.12	46.28 ± 5.95	29/18	26/21	EA + CR (*n* = 47)	CR (*n* = 47)	BL31-34	42	2020.02–2022.05	a,c,d,e,g
Sun 2023 (35)	B	72.8 ± 7.63	70.6 ± 6.06	44.52 ± 8.40	46.56 ± 10.10	15/5	12/8	EA + CR (*n* = 20)	CR (*n* = 20)	BL31-34	40	2019.05–2022.03	a,c,d,f,g,h
Zong 2024 (15)	NR	3.74 ± 1.37	3.52 ± 1.23	46.30 ± 11.57	48.03 ± 10.32	16/14	15/15	EA + CR (*n* = 30)	CR (*n* = 30)	EX-B2, BL23, BL35, DU20	14	2019.10–2022.10	a,d
Hu 2024 (36)	NR	6.3 ± 1.8	6.0 ± 1.6	39 ± 3.3	39.5 ± 3.5	15/15	18/12	EA + CR (*n* = 30)	CR (*n* = 30)	BL31-34, DU23, DU4, ST28	56	2020.01–2021.12	a,c,d,g
Ye 2024 (37)	A10 B70	6.01 ± 1.02	6.11 ± 1.05	46.01 ± 5.12	45.95 ± 5.07	28/12	26/14	EA + CR (*n* = 40)	CR (*n* = 40)	BL31-34, BL35	42	2022.06–2023.12	a,c,d,f,g

UMN, Upper motor neuron injury; LMN, Lower motor neuron injury; A, Complete spinal cord injury; B, Incomplete spinal cord injury; NR, Not Reported; EA, Electroacupuncture; CR, conventional rehabilitation; a, 24 h incontinence frequency; b, 24 h maximum urine output; c, 24 h single urination volume; d, residual urine volume; e, bladder volume; f, Qmax; g, PdetQmax; h, BC.

### 3.2 Risk of bias

**Figure 2:**
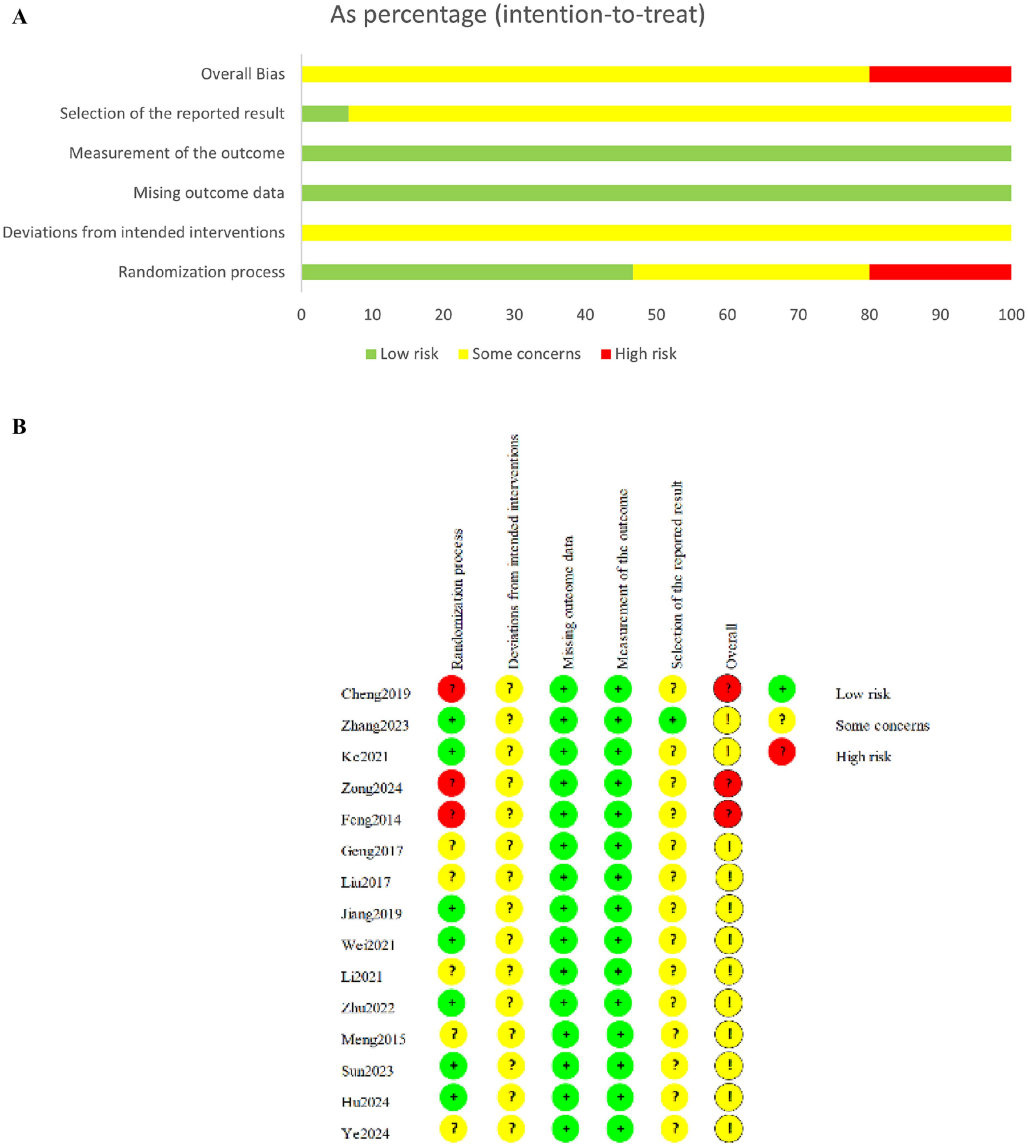
**(A)** Risk of bias graph. **(B)** Risk of summary.

Three studies (15, 26, 29) showed a high risk of bias, while the remaining studies exhibited some concerns. The randomization process was mentioned in all studies, nine articles used randomized table method and were rated as low risk, three articles sorted participants according to consultation order and admission time, and were rated as high risk, and three articles used unspecified grouping methods, leading to some concerns regarding potential selection bias. Due to the specific nature of acupuncture therapy, all studies were not blinded. However, the included outcome measures were less susceptible to bias from lack of blinding, thus deviations from intended interventions were assessed as low risk for all included studies. Additionally, none studies had registered protocols, leading to some concerns regarding biases in outcome confirmation and reporting (Figures 2A,B).

### 3.3 The forest of outcome index of urination diary

#### 3.3.1 24 h incontinence frequency

**Figure 3:**
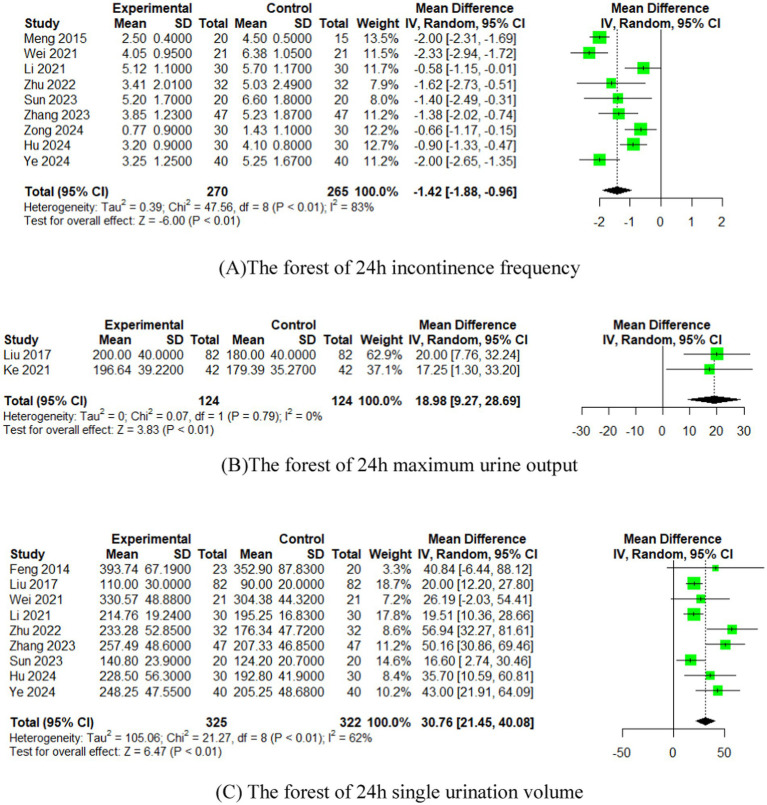
The forest of outcome index of urination diary. **(A)** The forest of 24 h incontinence frequency. **(B)** The forest of 24 h maximum urine output. **(C)** The forest of 24 h single urination volume.

In 9 studies (15–17, 32–37), the effect of EA (or combined with CR) on 24 h incontinence frequency was compared with that of CR. The meta-analysis showed that EA had superior effects compared to CR (*n* = 535, MD = −1.42, 95% CI (−1.88, −0.96), *p* < 0.01), with high heterogeneity (I^2^ = 83%) (Figure 3A).

#### 3.3.2 24 h maximum urine output

In 2 studies (28, 31), the effect of EA (or combined with CR) on 24 h maximum urine output was compared with that of CR. The meta-analysis indicated superior effects of EA over CR (*n* = 248, MD = 18.98, 95% CI (9.27, 28.69), *p* < 0.01), with low heterogeneity (I^2^ = 0%) (Figure 3B).

#### 3.3.3 24 h single urination volume

In 9 studies (16, 17, 26, 28, 32, 33, 35–37), the effect of EA (or combined with CR) on 24 h single urination volume was compared with that of CR. The meta-analysis demonstrated superior effects of EA compared to CR (*n* = 647, MD = 30.76, 95% CI (21.45, 40.08), *p* < 0.01), with moderate heterogeneity (I^2^ = 62%) (Figure 3C).

### 3.4 The forest of urodynamic outcome index

#### 3.4.1 Residual urine volume

**Figure 4:**
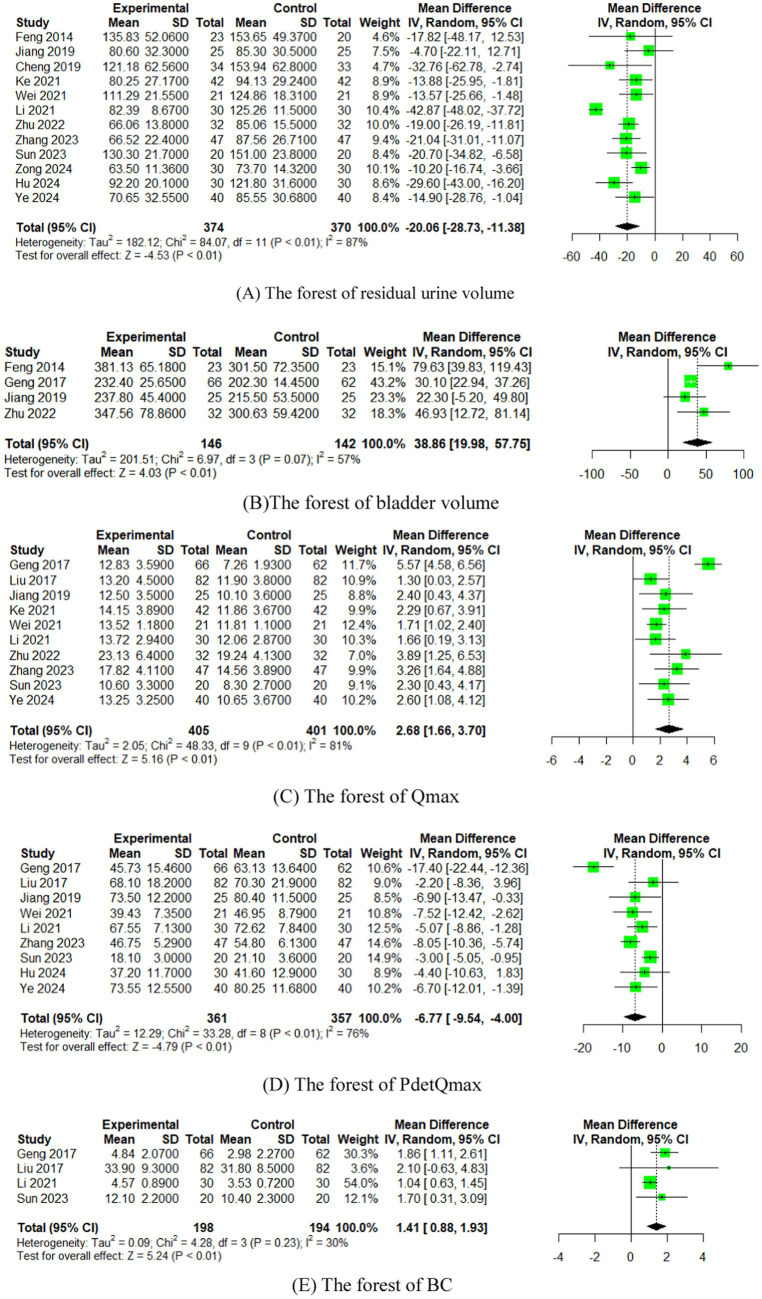
The forest of urodynamic outcome index. **(A)** The forest of residual urine volume. **(B)** The forest of bladder volume. **(C)** The forest of Qmax. **(D)** The forest of PdetQmax. **(E)** The forest of PdetQmax.

In 12 studies (15–17, 26, 29–33, 35–37), the effect of EA (or combined with CR) on residual urine volume was compared with that of CR. The meta-analysis showed that EA had superior effects compared to CR (*n* = 744, MD = −20.06, 95% CI (−28.73, −11.38), *p* < 0.01), with high heterogeneity (I^2^ = 87%) (Figure 4A).

#### 3.4.2 Bladder volume

In four studies (17, 26, 27, 30), the effect of EA (or combined with CR) on bladder volume was compared with that of CR. The meta-analysis indicated superior effects of EA over CR CR (*n* = 288, MD = 38.86, 95% CI (19.98, 57.75), *p* < 0.01), with moderate heterogeneity (I^2^ = 57%) (Figure 4B).

#### 3.4.3 Qmax

In 10 studies (16, 17, 27, 28, 30–33, 35, 37), the effect of EA (or combined with CR) on Qmax was compared with that of CR. The meta-analysis showed that EA had superior effects compared to CR (*n* = 806, MD = 2.68, 95% CI (1.66, 3.70), *p* < 0.01), with high heterogeneity (I^2^ = 81%) (Figure 4C).

#### 3.4.4 PdetQmax

In nine studies (16, 27, 28, 30, 32, 33, 35–37), the effect of EA (or combined with CR) on PdetQmax was compared with that of CR. The meta-analysis demonstrated superior effects of EA compared to CR (*n* = 718, MD = −6.77, 95% CI (−9.54, −4.00), *p* < 0.01), with high heterogeneity (I^2^ = 76%) (Figure 4D).

#### 3.4.5 BC

In four studies (27, 28, 32, 35), the effect of EA (or combined with CR) on BC was compared with that of CR. The meta-analysis indicated superior effects of EA over CR (*n* = 392, MD = 1.41, 95% CI (0.88, 1.93), *p* < 0.01), with low heterogeneity (I^2^ = 30%) (Figure 4E).

### 3.5 The sensitivity analysis of outcome index of urination diary

**Figure 5:**
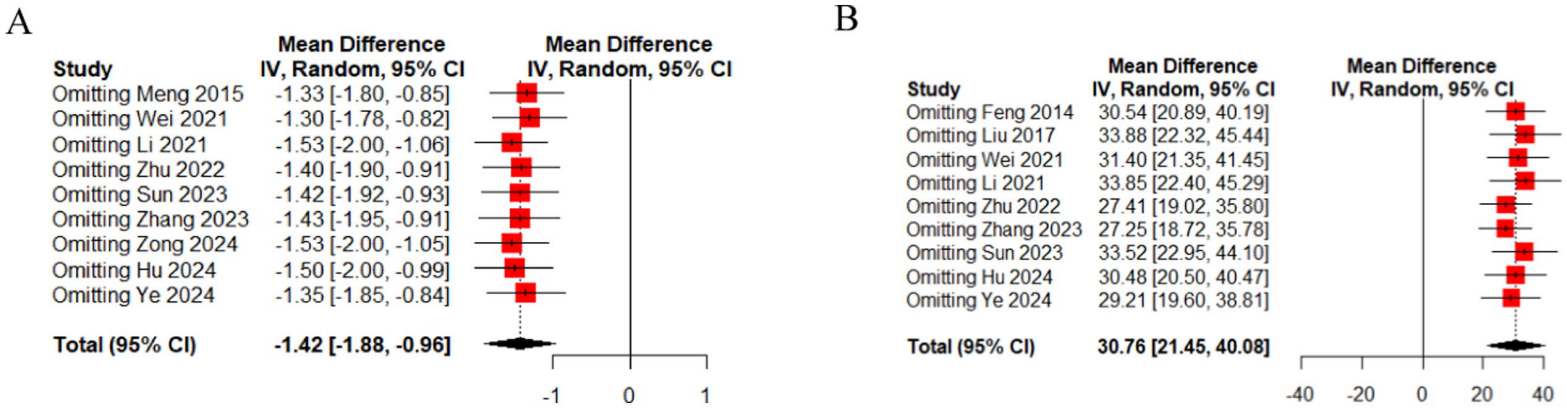
The sensitivity analysis of outcome index of urination diary [**(A)** 24 h incontinence frequency, **(B)** 24 h single urination volume].

We performed a leave-one-out sensitivity analysis by iteratively removing one study at a time. The point estimates remained within the 95% confidence interval (CI) of the complete analysis for the outcomes, namely 24 h incontinence frequency and 24 h single urination volume (Figure 5). This suggests that the results were stable.

### 3.6 The sensitivity analysis of urodynamic outcome index

**Figure 6:**
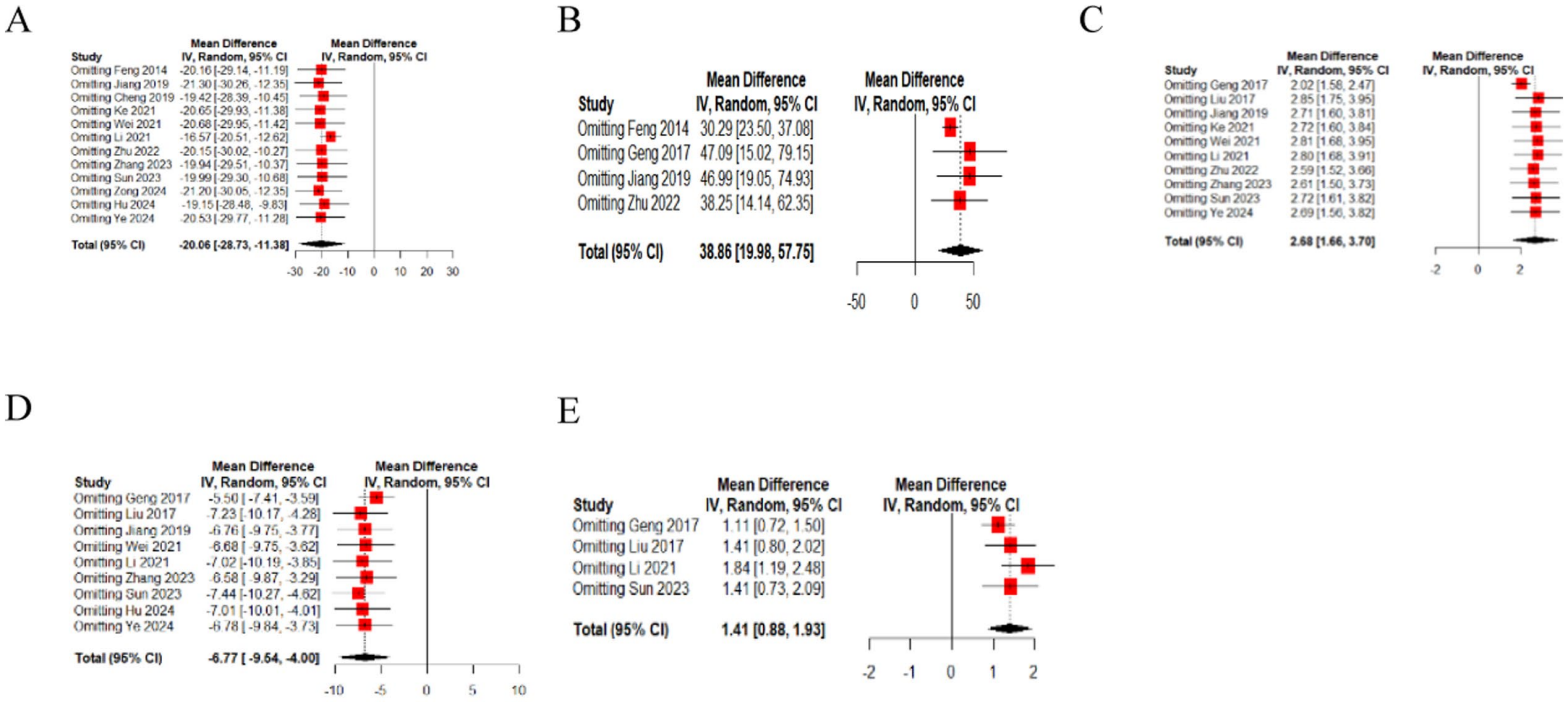
The sensitivity analysis of urodynamic outcome index [**(A)** residual urine volume, **(B)** bladder volume, **(C)** Qmax, **(D)** PdetQmax, **(E)** BC].

We performed a leave-one-out sensitivity analysis by iteratively removing one study at a time. The point estimates remained within the 95%CI of the complete analysis for the outcomes, which include residual urine volume, bladder volume, Qmax, PdetQmax, BC (Figure 6). This indicates that the results were stable.

### 3.7 Publication bias

**Figure 7:**
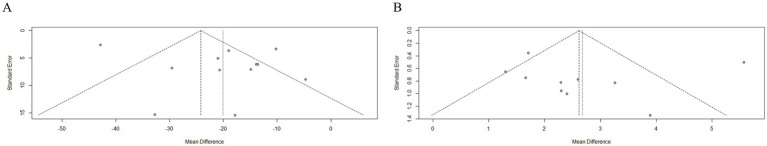
The funnel plot of urodynamic outcome index [**(A)** residual urine volume, **(B)** Qmax].

Eggers tests were performed to detect publication bias when more than 10 studies with the same outcome were included in the analysis. Since Residual urine volume and Qmax indicators exceeded this threshold, a funnel plot and Egger’s tests could be made to assess publication bias (Figure 7). After Egger’s tests, the *p*-values of the two were 0.2096 and 0.8085 respectively, so there was no risk of bias in either of them.

### 3.8 GRADE evidence profile for the studies in the meta-analysis

We extracted all relevant outcomes reported in the 15 included RCTs, specifically 24 h incontinence frequency, 24 h maximum urine output, 24 h single urination volume residual urine volume, bladder volume, Qmax, PdetQmax, BC. The GRADE analysis results indicated that the overall quality of evidence for various outcome indicators ranged from low to moderate, which was not conducive to our recommendation of the results. The reasons for downgrading were clarified with superscripts for each outcome (Table 2).

**Table 2:** GRADE evidence profile for the studies in the meta-analysis.

Outcome	No. study	No. patients	Certainty assessment					Summary of findings	
			Risk of bias	Inconsistency	Indirectness	Imprecision	Publication bias	Effect size Pooled MD (95% CI)	Certainty
24 h incontinence frequency	9	535	Serious^1^	Serious^2^	NS	NS	NA	−1.42, [−1.88, −0.96]	Low
24 h maximum urine output	2	248	NS	NS	NS	Serious^3^	NA	18.98, [9.27, 28.69]	Moderate
24 h single urination volume	9	647	Serious^1^	NS	NS	NS	NA	30.76, [21.45, 40.08]	Moderate
residual urine volume	12	744	Serious^1^	Serious^2^	NS	NS	NA	−20.06, [−28.73, −11.38]	Low
bladder volume	4	288	Serious^1^	NS	NS	Serious^3^	NA	38.86, [19.98, 57.75]	Low
Qmax	10	806	NS	Serious^2^	NS	NS	NA	2.68, [1.66, 3.70]	Moderate
PdetQmax	9	718	NS	Serious^2^	NS	NS	NA	−6.77, [−9.54, −4.00]	Moderate
BC	4	352	NS	NS	NS	Serious^3^	NA	1.41, [0.88, 1.93]	Moderate

^1^All the included studies were judged by Cochrane risk of bias tool version 2.0. All were judged as some concerns. ^2^The important heterogeneity was found. ^3^Number of participants were lower than 400. ^4^The 95% CI of the meta-analysis results overlapped with the invalid interval. ^5^Egger’s tests, *p* < 0.05 means publication bias (when the RCTs ≥ 10). No., number; CI, confidence interval; NS, not serious, NA, not available; MD, mean difference; VS, very serious.

### 3.9 The TSA analysis of outcome index of urination diary

**Figure 8:**
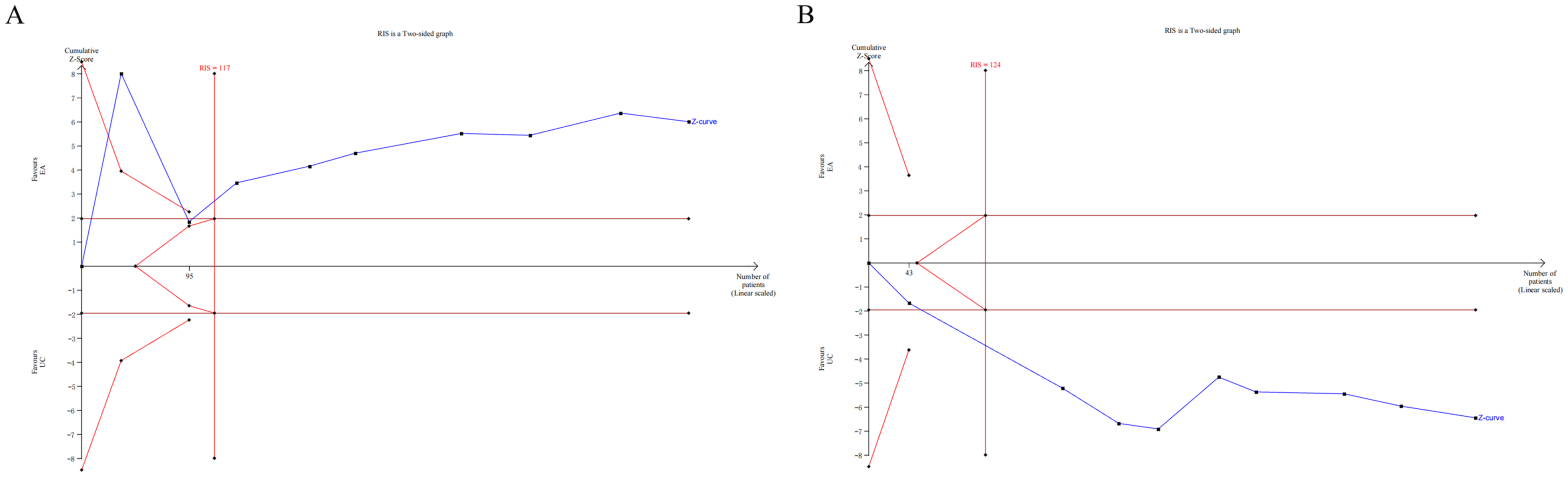
The TSA analysis of outcome index of urination diary [**(A)** 24 h incontinence frequency, **(B)** single urination volume].

The TSA was conducted for the 24 h incontinence frequency and single urination volume. Due to the relatively high heterogeneity and potential bias in the trials, a random-effect model (BT) was employed (37). The TSA plots for EA (alone or with CR) versus CR showed that Z-curve crossed both the trial sequential monitoring boundary and conventional monitoring boundary, and surpassed the required information size (RIS) axis. This indicates conclusive evidence for the efficacy of EA in improving incontinence in patients with UI (Figure 8).

### 3.10 The TSA analysis of urodynamic outcome index

**Figure 9:**
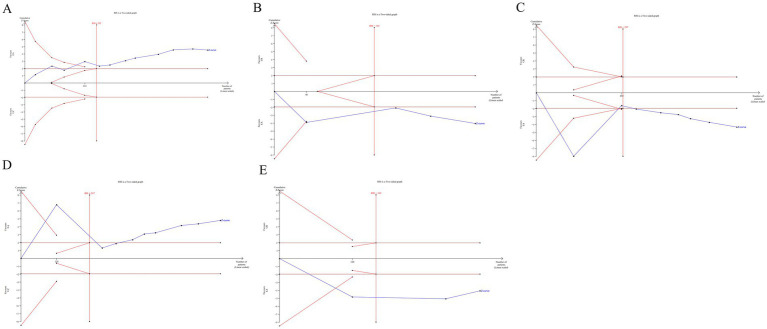
The TSA analysis of urodynamic outcome index [**(A)** residual urine volume, **(B)** bladder volume, **(C)** Qmax, **(D)** PdetQmax, **(E)** BC].

The TSA was performed for the outcomes of residual urine volume, bladder volume, Qmax, PdetQmax, BC. The TSA plots for EA (alone or with CR) versus CR showed that Z-curve crossed both the trial sequential monitoring boundary and conventional monitoring boundary, and surpassed the RIS axis. This confirms conclusive evidence for the efficacy of EA in improving urodynamic outcomes in patients with UI (Figure 9).

## 4 Discussion

### 4.1 Main findings

To the best of our knowledge, the effectiveness of EA for UI following SCI remains controversial. This meta-analysis is the first to evaluate the clinical efficacy of EA for UI. We included 15 studies in which all showed that EA (alone or with conventional rehabilitation, CR) was more effective for UI recovery compared to CR alone. This review indicates that EA has significant potential to enhance the recovery of UI in patients with SCI. Specifically, EA significantly reduces the frequency of 24 h urinary incontinence, increase the maximum 24 h urinary output and improves single urinary output. It effectively reduces the residual urine volume in the bladder, increases bladder capacity, and enhances maximum urinary flow rate. Moreover, EA decreases detrusor muscle pressure, improves bladder compliance, and overall bladder function.

A reduction of 1.42 episodes of urinary incontinence within 24 h has achieved the minimal clinically important difference (MCID). The Clinical Practice Guidelines for Comprehensive Management of Neurogenic Bladder^1^ suggest that a reduction of ≥1 episode of 24-h urinary incontinence can lower the risk of urinary tract infections and improve quality of life. According to the consensus on the management of overactive bladder (OAB) (38), an increase in single voided volume by ≥30 mL may result in a ≥25% reduction in daily voiding frequency (e.g., from 10 times to 7–8 times). Improved voiding efficiency can reduce post-void residual volume and decrease the risk of urinary tract infections. The European Association of Urology (EAU) recommends an increase in Qmax by ≥2 mL/s as a clinically significant indicator of relief from urethral obstruction. This suggests reduced bladder outlet resistance and enhanced urinary flow rate. An increase in Qmax reflects improved urethral sphincter coordination and enhanced detrusor contractility. PdetQmax ≥5 cm H₂O reduction indicates improved synchronized detrusor contractions and optimized urethral resistance during voiding. If PdetQmax decreases while Qmax increases, it suggests a significant improvement in detrusor-urethral coordination. The combined improvement of these parameters can predict effective upper urinary tract protection (39). Adverse reactions to EA were generally mild, primarily including bleeding at needle sites, numbness or soreness. Despite the very low to moderate certainty of evidence due to poor methodological quality and significant heterogeneity among studies, this review synthesizes the existing RCT evidence regarding the effect of EA on UI after SCI. EA may be a valuable addition to treatment protocols for UI following SCI and warrants integration into clinical guidelines.

SCI can disrupt the neural pathways between the bladder and the brain, resulting in a loss of voluntary control over the urination process; SCI may lead to either hyperreflexia or hyporeflexia of the bladder, commonly manifesting as detrusor overactivity (hyperactive detrusor muscle), which results in urgency and incontinence (40). Furthermore, SCI can affect the balance between the sympathetic and parasympathetic nervous systems, leading to dysfunction of the bladder and urethra. It may also cause changes in the bladder wall, including reduced capacity and compliance, ultimately resulting in incontinence (41).

Electroacupuncture (EA) can influence the activity of the sympathetic and parasympathetic nervous systems by stimulating relevant acupoints (42). The parasympathetic nervous system, in particular, plays a crucial role in promoting bladder emptying. Through EA treatment, the activity of the parasympathetic nerves can be regulated to control the relaxation and contraction of the bladder’s smooth muscles, thereby alleviating symptoms of incontinence. EA therapy can also modulate the release of various neurotransmitters, such as serotonin (5-HT), norepinephrine, and opioid substances (43), which in turn regulate neural activity and bladder function. These neurotransmitters are essential for the transmission of neural signals and the coordinated control of bladder muscles. Additionally, EA may reduce the release of inflammatory factors and modulate immune functions, thereby decreasing inflammatory responses and aiding in the management of incontinence (44). Studies have shown that EA can promote the regeneration and repair of nerve cells (45, 46). By stimulating the spinal cord and related neural regions, EA has the potential to foster the regeneration of nerve cells and the reconnection of synapses, thus partially restoring urinary control functions. This systematic review aims to comprehensively evaluate the research evidence on EA for UI following SCI. It assesses the clinical efficacy and safety of EA and provides a reference for clinical practice.

### 4.2 Quality summaries

Three studies exhibited a high risk of bias due to incorrect randomization method. Variations in acupuncture treatment protocols, practitioner techniques, and the type of SCI (with unclear grouping by injury degree and segment) could contribute to the observed heterogeneity in therapeutic outcomes. None of the included studies implemented blinding due to the nature of electroacupuncture, potentially causing implementation bias. The studies did not specify whether outcome measurements were conducted by an independent third party, raising the possibility of measurement bias if performed by the same physician administering EA. Additionally, none of the studies mentioned pre-registration in the clinical trial registry, making it difficult to ascertain if all intended outcomes were reported, thus introducing reporting bias. The GRADE analysis results indicate that the overall quality of evidence across outcome indicators ranges from low to moderate, which was not conducive to our recommendation of the results.

### 4.3 Outlook and recommendations

Future RCTs should adhere to the STRICTA and CONSORT guidelines, clearly describe random number generation and allocation concealment, and pre-registration trial protocol. Moreover, outcome assessors, participants, and physicians should be blinded, and any adverse effects should be clearly documented. Improved methods for assessing the effectiveness of blinding in acupuncture RCTs are needed. Bang et al. (47) developed a high-quality blinding assessment tool for clinical trials, which should be widely adopted in the future.

### 4.4 Strengths and limitations

This paper presents the first meta-analysis on the efficacy of EA for treating UI following SCI, thereby filling a significant research gap. TSA analysis was employed to validate the robustness of the research on multiple outcome indicators, minimizing false positives. However, several limitations should be considered. Firstly, the overall methodology and reporting quality of the included studies were poor, affecting the credibility of the results. Secondly, significant heterogeneity among the studies impacted the meta-analysis findings. Moreover, the limited published literature precluded a comprehensive analysis of the long-term efficacy of EA. Moreover, the studies included were predominantly domestic, with few international reports, reducing the applicability of the findings. Future high-quality RCTs are necessary to provide a reliable basis for using EA to treat UI after SCI. Lastly, the search strategy primarily focused on incontinence. All the qualifying studies involved patients with upper motor neuron injury, which is commonly associated with overactive bladder. Future literature reviews should expand the search terminology to include “overactive bladder” and/or “upper motor neuron bladder” to achieve a more comprehensive evaluation of incontinence and assess the efficacy of EA in populations with SCI.

## 5 Conclusion

EA can significantly reduce the frequency of 24 h urinary incontinence, increase both maximum 24 h urinary output and single urinary output. It effectively reduces the residual urine volume in the bladder, increases bladder capacity, improves maximum urinary flow rate, decreases detrusor muscle pressure, and enhances bladder compliance. EA shows great potential for improving bladder function control in patients with UI following SCI. However, this study has some limitations and additional high-quality RCTs are required to validate these findings.

## Data Availability

The original contributions presented in the study are included in the article/Supplementary material, further inquiries can be directed to the corresponding author.

## Footnotes

^1^ https://rs.yiigle.com/cmaid/1526081
