# Supplementary material for: Clinical efficacy of electroacupuncture for urinary incontinence following spinal cord injury: a meta-analysis and trial sequential analysis
Source: Front Neurol. 2025 Sep 26;16:1573090. doi: 10.3389/fneur.2025.1573090 (PMC12512171; doi:10.3389/fneur.2025.1573090)
Supplement: Supplementary file 1 [file Data_Sheet_1.pdf]

Database	
CNKI	SU%=(‘电针’ + ‘电刺激’)*(‘脊髓损伤’ + ‘脊髓挫伤’ + ‘创伤性脊髓病’ + ‘脊髓创伤’ + ‘脊髓横断伤’)*(‘尿失禁’ + ‘神经源性膀胱’ + ‘神经源性膀胱功能障碍’ + ‘无抑制性神经源性膀胱’ + ‘失张力神经源性膀胱’ + ‘痉挛性神经源性膀胱’)
Wanfang	主题:((“电针” or “电刺激”) and (“脊髓损伤” or “脊髓挫伤” or “创伤性脊髓病” or “脊髓创伤” or “脊髓横断伤”) and (“尿失禁” or “神经源性膀胱” or “神经源性膀胱功能障碍” or “无抑制性神经源性膀胱” or “失张力神经源性膀胱” or “痉挛性神经源性膀胱”))
VIP	M=((“电针” or “电刺激”) and (“脊髓损伤” or “脊髓挫伤” or “创伤性脊髓病” or “脊髓创伤” or “脊髓横断伤”) and (“尿失禁” or “神经源性膀胱” or “神经源性膀胱功能障碍” or “无抑制性神经源性膀胱” or “失张力神经源性膀胱” or “痉挛性神经源性膀胱”))
CBM	((“电针” or “电刺激”) and (“脊髓损伤” or “脊髓挫伤” or “创伤性脊髓病” or “脊髓创伤” or “脊髓横断伤”) and (“尿失禁” or “神经源性膀胱” or “神经源性膀胱功能障碍” or “无抑制性神经源性膀胱” or “失张力神经源性膀胱” or “痉挛性神经源性膀胱”))
PubMed	((electroacupuncture OR “Electro-stimulation” [Mesh]) AND (“Spinal cord injury” OR “Post-traumatic myelopathy” OR “Spinal cord contusion” OR “Traumatic myelopathy” OR “Spinal cord trauma” OR “Traumatic spinal cord injury” OR “Transverse spinal cord injury” [Mesh])) AND (“Neurogenic bladder” OR “Neurogenic bladder dysfunction” OR “Non-inhibitory neurogenic bladder” OR “Dystonic neurogenic bladder” OR “Spasticity neurogenic bladder” OR “Urinary incontinence” [Mesh])
Embase	('electroacupuncture': ab,ti OR 'electro-stimulation ': ab.ti) AND ('Spinal cord injury': ab,ti OR 'Post-traumatic myelopathy': ab,ti 'Spinal cord contusion': ab,ti OR 'Traumatic myelopathy': ab,ti OR 'Spinal cord trauma': ab,ti OR 'Traumatic spinal cord injury': ab,ti OR 'Transverse spinal cord injury': ab.ti)AND ('Neurogenic bladder': ab,ti OR 'Neurogenic bladder dysfunction': ab,ti OR 'Non-inhibitory neurogenic bladder': ab,ti OR 'Dystonic neurogenic bladder': ab,ti OR 'Spasticity neurogenic bladder': ab,ti OR 'Urinary incontinence': ab,ti)
Cochrane Library	#1 (electroacupuncture OR “Electro-stimulation”): ti,ab,kw<br>#2 (“Spinal cord injury” OR “Post-traumatic myelopathy” OR “Spinal cord contusion” OR “Traumatic myelopathy” OR “Spinal cord trauma” OR “Traumatic spinal cord injury” OR “Transverse spinal cord injury”): ti.ab.kw<br>#3(“Neurogenic bladder” OR “Neurogenic bladder dysfunction” OR “Non-inhibitory neurogenic bladder” OR “Dystonic neurogenic bladder” OR “Spasticity neurogenic bladder” OR “Urinary incontinence”): ti,ab,kw<br>#1 and #2 and #3
Web of Science	TS= ((‘electroacupuncture’ OR ‘Electro-stimulation’) AND (‘Spinal cord injury’ OR ‘Post-traumatic myelopathy’ OR ‘Spinal cord contusion’ OR ‘Traumatic myelopathy’ OR ‘Spinal cord trauma’ OR ‘Traumatic spinal cord injury’ OR ‘Transverse spinal cord injury’) AND (‘Neurogenic bladder’ OR ‘Neurogenic bladder dysfunction’ OR ‘Non-inhibitory neurogenic bladder’ OR ‘Dystonic neurogenic bladder’ OR ‘Spasticity neurogenic bladder’ OR ‘Urinary incontinence’))

Table Sl. Search strategies.

CBM: China Biology Medicine; CNKI: China National Knowledge Infrastructure; Embase: Excerpta Medica Database; VIP: VIP Database for Chinese Technical Periodicals.
